# Development of a robust FT-IR typing system for *Salmonella enterica*, enhancing performance through hierarchical classification

**DOI:** 10.1128/spectrum.00159-25

**Published:** 2025-05-27

**Authors:** Diego Fredes-García, Javiera Jiménez-Rodríguez, Alejandro Piña-Iturbe, Pablo Caballero-Díaz, Tamara González-Villarroel, Fernando Dueñas, Aniela Wozniak, Aiko D. Adell, Andrea I. Moreno-Switt, Patricia García

**Affiliations:** 1 Escuela de Medicina Veterinaria, Facultad de Medicina, Pontificia Universidad Católica de Chile 28033, Santiago, Chile; 2 Departamento de Laboratorios Clínicos, Escuela de Medicina, Facultad de Medicina, Pontificia Universidad Católica de Chile 28033, Santiago, Chile; 3 Red de Salud UC-CHRISTUS, Laboratorio de Microbiologia 28033, Santiago, Chile; 4 Escuela de Medicina Veterinaria, Centro de Estudios e Investigación en Salud y Sociedad, Facultad de Ciencias Médicas, Universidad Bernardo O’Higgins 28033, Santiago, Chile; 5 Escuela de Medicina Veterinaria, Facultad de Ciencias de la vida, Universidad Andrés Bello 682708 , Santiago, Chile; Navarrabiomed-Universidad Pública de Navarra (UPNA)-Complejo Hospitalario de Navarra (CHN), IdiSNA, Pamplona, Navarra, Spain

**Keywords:** *Salmonella*, serotyping, FT-IR, rapid diagnostic, salmonellosis

## Abstract

*Salmonella enterica* is a leading cause of foodborne illnesses globally, with significant mortality rates, especially among vulnerable populations. Traditional serotyping methods for *Salmonella* are accurate but expensive, resource-intensive, and time-consuming, necessitating faster and more reliable alternatives. This study evaluates the IR Biotyper, a Fourier-transform infrared spectroscopy system, in differentiating *Salmonella* serovars. We assessed 458 isolates of nine *Salmonella* serovars (Infantis, Enteritidis, Typhimurium, I,4,[5],12:i:-, Montevideo, Agona, Thompson, Panama, and Abony) from diverse sources. The IR Biotyper was used to acquire spectra from these isolates. Machine learning algorithms, including support vector machines, were trained to classify the isolates. The accuracy of classifiers was validated using a validation set to determine sensitivity, specificity, positive predictive value, and negative predictive value. Initial classifiers showed high accuracy for Abony, Agona, Enteritidis, and Infantis serovars, with sensitivities close to 100%. However, classifiers for *S*. Typhimurium, *S*. Panama, and *S*. Montevideo exhibited lower performance. Implementing a hierarchical classification system enhanced the accuracy of serogroup O:4 serovars, demonstrating that this approach offers a robust framework for *Salmonella* serovar identification. The hierarchical system enables progressive refinement of classification, minimizing misclassifications by focusing on serogroup-specific features, making it adaptable to complex data sets and diverse serovars. The IR Biotyper demonstrates high potential for rapid and accurate *Salmonella* serovar identification. This study supports its implementation as a cost-effective, high-throughput tool for pathogen typing, enhancing real-time epidemiological surveillance, and guiding treatment strategies for salmonellosis. This method establishes a robust and scalable framework for advancing *Salmonella* serotyping practices across clinical, industrial, and public health domains by leveraging hierarchical classification.

**IMPORTANCE:**

Early and accurate identification of *Salmonella* serovars is extremely important for epidemiological surveillance, public health, and food safety. Traditional serotyping is very successful but is laborious and costly. In this study, we demonstrate the promise of Fourier-transform infrared spectroscopy together with machine learning as a means for *Salmonella* serotyping. Using hierarchical classification, we attain optimal serovar identification accuracy, particularly for challenging-to-type serogroups. Our findings recognize the IR Biotyper as a high-throughput, scalable pathogen typing solution that offers real-time data that can enable enhanced outbreak response and prevention of foodborne disease. The approach bridges the gap between traditional microbiological practice and sophisticated analytical technology, the path to more effective, cost-saving interventions in the clinical, industrial, and regulatory settings. Application of these technologies can significantly improve *Salmonella* surveillance-control and Public Health outcomes.

## INTRODUCTION

*Salmonella enterica* is one of the major causes of foodborne disease, causing more than 420,000 deaths worldwide, in 2019 only (1). While salmonellosis commonly presents as self-limited gastroenteritis, children under 5 years, adults over 65 years, and immunocompromised people are at a higher risk of developing a life-threatening severe invasive form of the disease which requires the use of third-generation cephalosporins, fluoroquinolones, or azithromycin as first-line antibiotics (2). The *Salmonella* genus has two species: *S. enterica* and *Salmonella bongori*. Within *S. enterica,* there are six subspecies; however, most human infection is caused by *S. enterica* subsp. *enterica* (3). Serological typing of the lipopolysaccharide (O) antigen results in more than 40 *Salmonella* serogroups, which, when phase 1 (H1) and phase 2 (H2) flagellar antigens are included, classify *Salmonella* in more than 2,600 serovars, according to the White-Kauffmann-Le Minor scheme (4, 5). Nevertheless, most human cases are caused by only a few serovars, such as *S*. Enteritidis and *S*. Typhimurium in Europe and the United States, where they are responsible for 30%-65% of *Salmonella* infections, for example (6, 7). Moreover, different lineages of these and other less frequent/emerging serovars (e.g., Infantis, Newport, Derby, and Heidelberg, among others) are constantly causing outbreaks of foodborne disease and have acquired antimicrobial resistance determinants against third-generation cephalosporins and fluoroquinolones, limiting the treatment options in cases of severe infection (6–12). Therefore, fast and reliable methods for identifying *Salmonella* serovars are required for performing real-time surveillance of high-risk/high-burden serovars within a One Health framework, that is, in humans, animals, and the environment, as control of human infections caused by this pathogen is influenced by its presence in animals and the environment (13).

Currently, the gold standard for *Salmonella* serovar identification is phenotypic serotyping using the White-Kauffmann-Le Minor scheme and antisera targeting the O, H1, and H2 antigens. However, this method requires more than 150 specific antisera and well-trained experienced personnel (14). Moreover, if only one H antigen is detected, additional steps are performed to confirm whether the tested strain is monophasic (has only one H antigen) or not. Therefore, traditional serotyping is usually carried out in reference laboratories and takes several days to produce results (14, 15). As alternatives to traditional serotyping, molecular methods based on detecting serovar-specific genetic markers are also available and can produce results in a reduced time (16). However, these methods require expensive reactions, additional steps of DNA extraction, and/or purification besides bacterial isolation and are focused on a limited number of relevant/common serovars (16, 17). Whole-genome sequencing (WGS) coupled with *in silico* serovar prediction has become a powerful tool for *Salmonella* serotyping, as it provides highly accurate typing results and supplies the raw material (the genome sequence) for further in-depth characterization of the pathogen (16, 18). Nevertheless, there are multiple barriers to the routine implementation of WGS in the clinical setting, especially for low-middle-income countries, including high setup and running costs, a lack of studies addressing the cost-effectiveness of WGS implementation, and logistic challenges about genomic data storage, analysis, and reporting (19). Cost-effective, high-throughput serotyping alternatives that can produce results in less than 24 hours are desirable.

The Fourier-transform infrared (FT-IR) spectroscopy is a vibrational spectroscopic technique that can be applied to assess the chemical composition of a sample (20). The different vibrational bond energies between the atoms in the sample molecules absorb IR radiation at different wavelengths, producing a spectrum characteristic of their composition (21). With multiple biological/biomedical applications, such as cell imaging, cancer detection, food safety, toxicology, microbiology, and others (21), FT-IR spectroscopy is considered a versatile, fast, and low-cost technique (22). For bacterial typing, the spectra obtained by FT-IR spectroscopy can work as fingerprints that allow differentiation of bacteria at the species, subspecies, and even serogroup/serotype level (23–25), and its application for strain clonality studies, outbreak investigation, and bacterial identification is being studied, as it can provide results in less than 4 hours at a fraction of the cost of higher resolution techniques such as WGS (15, 26–29). The IR Biotyper (Bruker Daltonics GmbH & Co. KG, Germany) is an IR spectrometer coupled to a microplate compartment, which, together with the OPUS software, allows the acquisition and analysis of FT-IR spectra for multiple bacterial samples (30). The IR Biotyper can be trained with different bacteria with the aid of machine-learning algorithms to enable the typing of unknown samples. Consequently, the implementation of the IR Biotyper, as a low-cost/high-throughput tool for pathogen typing in the clinical microbiology laboratory, has been proposed (26, 27). Using this approach, the discrimination of clinically relevant *Salmonella* serogroups has been achieved with high accuracy (15). However, FT-IR-based typing of this pathogen at the serovar level is still challenging, especially when multiple serovars of the same serogroup are tested (15, 31). To overcome this difficulty, a step-wise approach for serovar classification has been proposed, in which the step of serovar classification should follow the first step of serogroup classification; however, this approach has not yet been evaluated (32). In this study, we tested the capabilities of the IR Biotyper using the step-wise approach for the classification of the major *Salmonella* serovars found in the clinical, environmental, and food/avian niches in Chile.

## MATERIALS AND METHODS

### Bacterial collection, culture media, and conditions

A total of 458 well-characterized isolates of *S. enterica* (corresponding to nine serovars) corresponding to *Salmonella* Infantis (*n* = 163), *Salmonella* Enteritidis (*n* = 117), *Salmonella* Typhimurium (*n* = 51), *Salmonella* I,4,[5],12: i:- (*n* = 16), *Salmonella* Montevideo (*n* = 6), *Salmonella* Agona (*n* = 63), *Salmonella* Thompson (*n* = 14), *Salmonella* Panama (*n* = 16), and *Salmonella* Abony (*n* = 12) were used in this study (Table S1).

The isolates were provided by different institutions and investigation centers and were obtained from different sources, such as environmental water (*n* = 277) (33), swine stool (*n* = 1) (34), an equine hospital facility (*n* = 9) (35), a backyard poultry farm (*n* = 8) (36), poultry meat (*n* = 35) (12), and clinical human samples (*n* = 12) (37) from patient consultant at Red de Salud UC-CHRISTUS (*n* = 114). Permission to use the human clinical isolates was granted by the Comité Ético Científico de Ciencias de la Salud UC (Protocol ID 240621037).

In summary, the isolation and serotyping protocol were conducted by processing samples through traditional microbiological techniques. Strain confirmation was conducted using molecular methods as outlined by Toro et al. (33). Serovar identification was performed through the standard Kaufmann-White-LeMinor agglutination system, complemented by molecular approaches (36, 38), as well as *in silico* analysis based on WGS. Table S1 provides detailed information on the collection year, source, and methodology for each isolate.

### Sample preparation

Strains were stored in 12%–20% glycerol stocks at −80°C before they were retrieved on trypticase soy agar (TSA, Biomerieux, France) and passed at least two times before subculturing the samples in TSA; each incubation was performed at 37 ± 2°C for 24 hours. The sample preparation adhered to the manufacturer’s guidelines. Briefly, a 1-µL overloaded loop of bacterial colonies was taken from the densely populated area of the culture and suspended on 50 µL of a 70% ethanol solution within an IR Biotyper suspension vial. After vortexing, 50 µL of deionized water was introduced, and the mixture was thoroughly mixed by vortexing. Next, 15 µL of the bacterial suspension was dispensed in three separate technical replicates onto the 96-spot silicon IR Biotyper target and left to dry for 15–20 minutes at 35 ± 2°C. For all isolates categorized, measurements were taken in three or more distinct biological replicates to ensure a more equitable number of spectra for each serovar. As for the quality control, the Infrared Test Standards (IRTS 1 and IRTS 2) provided in the IR Biotyper kit were resuspended in 100-µL deionized water, and 60 µL of absolute ethanol was added and thoroughly mixed. A 10-µL suspension was then deposited in duplicate onto the IR Biotyper target and allowed to dry following the same procedure as the samples.

### Spectra acquisition and analysis

Acquisition of spectra was conducted in the UC-Christus Clinical Microbiology laboratory. This process involved transmission mode within the spectral range of 3,996–500 cm-1 (mid-IR), utilizing the IRBT spectrometer and OPUS software version 8.2.28 from Bruker Optics GmbH & Co. KG. Spectra processing and visualization was executed using the IR Biotyper Client Software from Bruker Daltonics, specifically version 4.0, with default settings recommended by the manufacturer. Before acquiring sample spectra, quality control measurements (IRTS 1 and IRTS 2) were conducted in each run. For each isolate, we obtained at least three spectrums, and to ensure quality, some spectra were reprocessed to achieve three correct measurements, obtaining a total of 5,428 spectra for the training and validation steps.

### Machine learning and development of automated classifiers

The strategy of classification was divided into steps. The first step corresponds to the determination of the “O” antigen (O:4, O:7, and O:9) using the “*Salmonella* serogroup” classifier of the IR Biotyper Client Software from Bruker Daltonics (version 4.0), and the second step performs the serovar classification. Machine learning (ML) algorithm support vector machine (SVM) was utilized to construct the serovar IR-BT automated classifiers. The ML undergoes training with a set of well-characterized isolates to identify the specific characteristics of each serovar, and ML algorithm learns to recognize knowns, obtaining a real-time classification.

Accuracy was automatically calculated by the IR Biotyper software using a confusion matrix. The matrix determines the class recall as the percentage of correctly predicted spectra among all true spectra, considering uncertain spectra as errors. The class precision is calculated as the ratio of true positive cases to the sum of true positive and false positive cases, corresponding to the positive predictive value (PPV). Additionally, overall accuracy is computed as the proportion of all true positive cases relative to the total number of cases. Sensitivity is defined as the ratio of true positive cases to the sum of true positive and false negative cases, excluding uncertain cases. Specificity is calculated by dividing the number of true negative cases by the sum of true negative and false positive cases. Meanwhile, the negative predictive value (NPV) is determined as the ratio of true negative cases to the sum of true negative and false negative cases.

To better understand the clustering performance of the serovars, we performed a linear discriminant analysis (LDA) using IR Biotyper Client software version 4.0. The parameters of the classification algorithms, such as the number of principal components (PCs) and the C-value or cost value, which evaluate the penalty associated with misclassification errors made by the model during training, were optimized through a trial-and-error process for maximum accuracy. The classifier version that produced the best results was selected and further validated with an external testing set to ensure robustness and reliability (32). The steps described above were performed using the IR Biotyper software version 4.0. The variance of each LDA was calculated by the software.

For more detailed information about the equipment software, visit https://www.bruker.com/en/applications/microbiology-and-diagnostics/food-beverage-microbiology/ir-biotyper-for-food-microbiology.html (30).

#### O-antigen classifier validation

To validate the O-antigen classifier integrated into the equipment, a comprehensive analysis was conducted using the total of isolates (*n* = 458) corresponding to the 5,428 spectra, which included representations from the serogroups O:4 (*S*. Typhimurium, *S*. I,4,[5],12:i:-, *S*. Abony, *S*. Agona), O:7 (*S*. Infantis, *S*. Montevideo, *S*. Thompson), and O:9 (*S*. Enteritidis, and *S*. Panama).

#### Serogroup O:4 training and validation

Serovars with more than five isolates per type were chosen. The proportion of training and validation depends on the heterogeneity within each group (O:4, O:7, O:9). The total isolates, together with their corresponding spectra, were divided into two randomized data sets, and the larger percentage was used for training and the remaining for validation.

For the first training set, we focused on serogroup O:4, which includes the serovars spectra *S*. Typhimurium (*n* = 583 and *S*. I,4,[5],12: i:- (*n* = 219), *S*. Agona (*n* = 889), and *S*. Abony (*n* = 103). Serovars *S*. Typhimurium and *S*. I,4,[5],12: i:- were used as a single target called *S*. Typhimurium/Monophasic (*n* = 802). Out of the total spectra obtained (*n* = 1,794), 80% (*n* = 1,460) were included in the training set (Table S1), these spectra belong to a subset of isolates (*n* = 109). The remaining 20% of the spectra (*n* = 334) were used for the validation set; these spectra belong to the remaining isolates (*n* = 33).

#### Serogroup O:7 training and validation

In the second round of training, we focused on serovars from serogroup O:7, including spectrums of the serovars *S*. Infantis (*n* = 1,681), *S*. Thompson (*n* = 200), and *S*. Montevideo (*n* = 45), generating a total of 1,926 new spectra. Of these new spectra, 58% (*n* = 1,113) were incorporated into the training set; these spectra belong to a subset of isolates (*n* = 104). The remaining 42% (*n* = 813) were added to the original validation data set; these spectra belong to the remaining isolates (*n* = 79).

#### Serogroup O:9 training and validation

In the third round of training, we focused on serovars from serogroup O:9, including *S*. Enteritidis (*n* = 1528) and *S*. Panama (*n* = 180). Out of a total of 1,708 spectra, 80% (*n* = 1,393) were used for classifier development; these spectra belong to a subset of isolates (*n* = 107), while the remaining 20% (*n* = 315) were included in the validation set; these spectra belong to the remaining isolates (*n* = 26).

## RESULTS

### O-antigen classifier validation

In the analysis of the total of 5,428 spectra, the O-antigen classifier validation achieved 100% accuracy, correctly predicting the serogroup of each one of the spectra; detailed accuracy values are provided in Table 1.

**TABLE 1:** Confusion matrix for the validation of O-antigen^*a*^

		Class predicted				
		O:4	O:7	O:9	Uncertain	Class recall
**Actual class**	Panama	0	0	180	0	100%
	Thompson	0	200	0	0	100%
	Typhimurium/1,4,[5],12: i:-	801	0	0	1	100%
	Abony	103	0	0	0	100%
	Agona	889	0	0	0	100%
	Enteritidis	0	0	1,528	0	100%
	Infantis	0	1,681	0	0	100%
	Montevideo	0	45	0	0	100%
	Class precision	100%	100%	100%		

^*a*^ Accuracy: 100% (5,427/5,428).

### Serogroup O:4 training and validation

**Fig 1:**
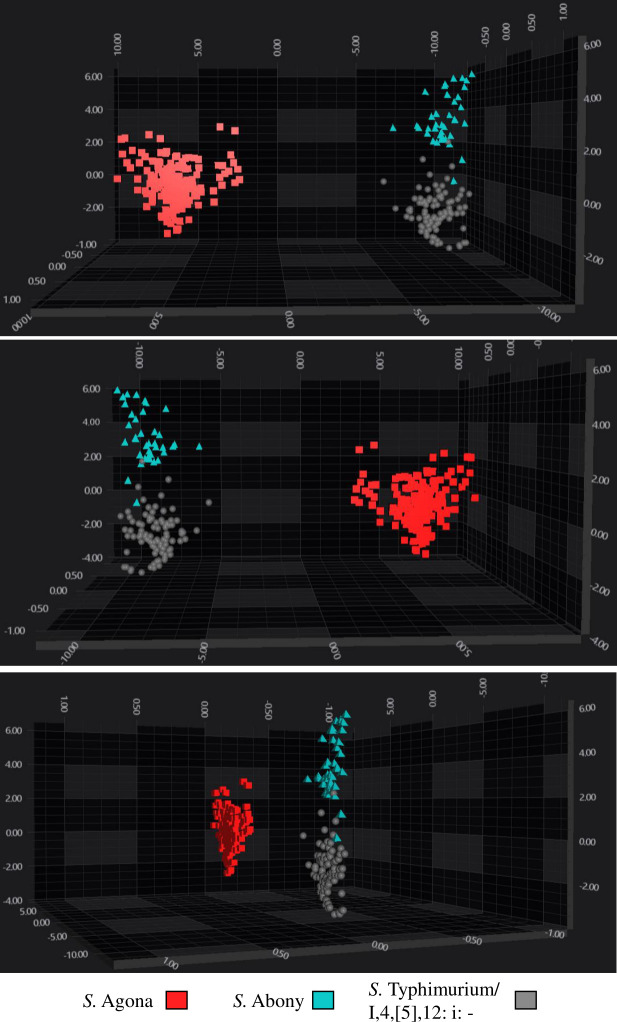
3D scatter plot of the third training performed, showing the distribution of serovars of Salmonella serogroup O:4 species in IR spectral space. A total of 30 PCs were used to create a LDA by serovar. The spectra are colored according to the serovars: red squares: *S*. Agona; cyan triangles: *S*. Abony; gray circles: *S*. Typhimurium/ I,4,[5],12:I:-.Every symbol represents a different isolate.

The ML algorithm used to construct the initial training model was a Library for Support Vector Machines with a Radial Basis Function (LIB_SVM_RBF). This process involved the spectral range of 1,300–800 cm^−1^ and was performed with PC30 and a C-value of 50 (Fig. 1). In total, 334 spectra were analyzed to perform the validation step. The automated classifiers for *S*. Abony showed the best performance (Table 2), achieving 100% sensitivity, specificity, PPV, and NPV, followed by the *S*. Agona and *S*. Typhimurium/1,4,[5], 12: i:- (Table 3). The LDA analysis showed that the isolates belonging to the Agona serovar exhibited a better clustering performance compared to the rest of the developed classifiers, followed by the serovar Abony and then *S*. Typhimurium/1,4,[5],12: i:-.

**TABLE 2:** Confusion matrix for the validation of O:4 classifier^*a*^

			Class predicted				
			Typhimurium/ 1,4,[5],12: i:-	Abony	Agona	Uncertain	Class recall
**Actual class**		Typhimurium/ 1,4,[5],12: i:-	89	0	2	5	93% (89/96)
		Abony	0	38	0	0	100% (38/38)
		Agona	0	0	198	2	99% (198/200)
		Class precision	100%	100%	99%		

^*a*^ Accuracy: 97% (325/334).

**TABLE 3:** Values of sensitivity, specificity, PPV, and NPV per serogroup O:4

	Sensitivity	Specificity	PPV	NPV
Typhimurium/ 1,4,[5],12: i:-	98%	100%	100%	99%
Abony	100%	100%	100%	100%
Agona	100%	98%	99%	100%

### Serogroup O:7 training and validation

**Fig 2:**
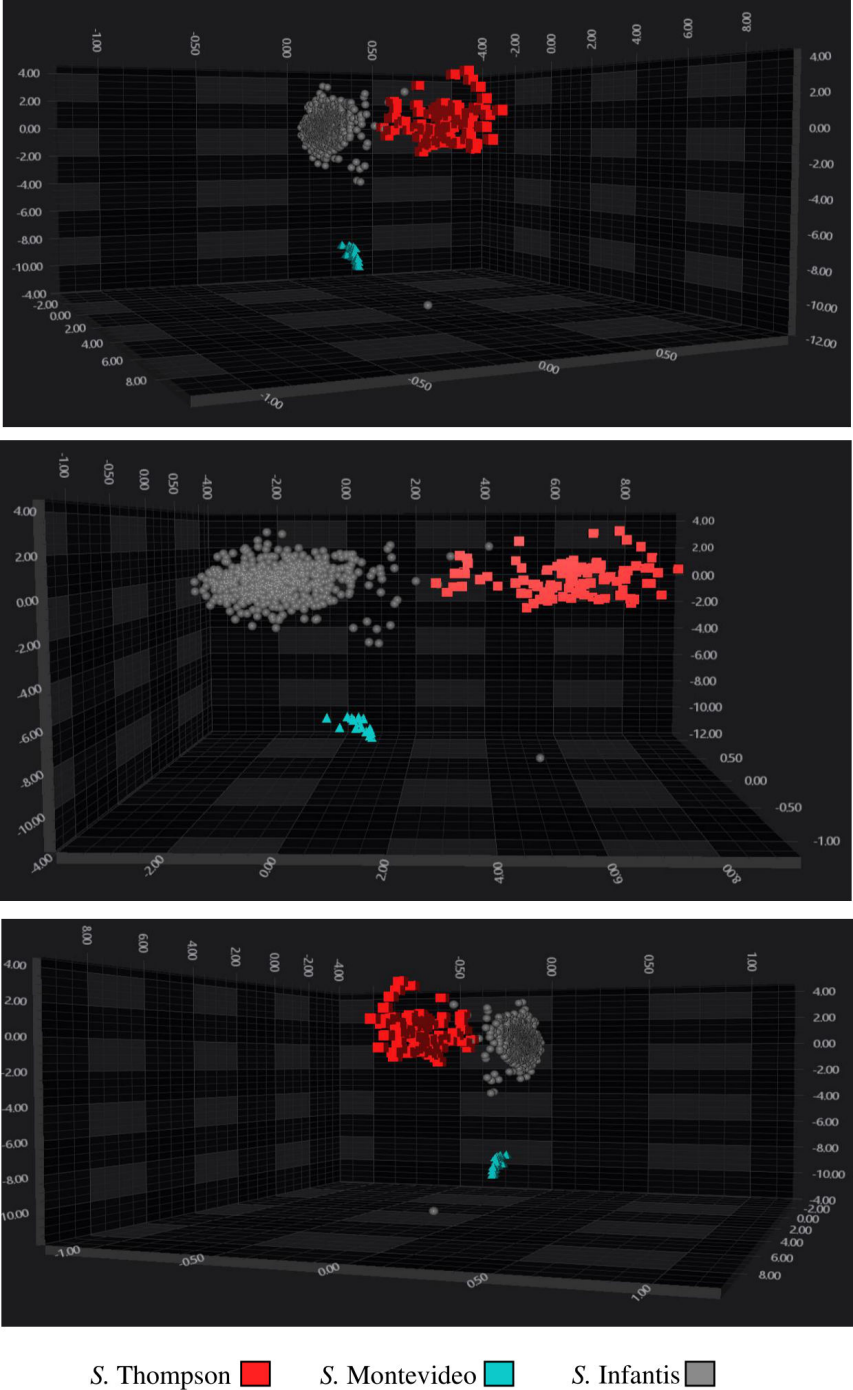
3D scatter plot of the third training performed, showing the distribution of serovars of *Salmonella* serogroup O:7 species in IR spectral space. A total of 30 PCs were used to create a LDA by serovar. The spectra are colored according to the serovars: red squares: *S*. Thompson; cyan triangles: *S*. Montevideo; gray circles: *S*. Infantis. Every symbol represents a different isolate.

The ML algorithm developed for this training was LIB_SVM_RBF, which involved a spectral range of 1,300–800 cm^−1^. SVM was performed with PC30 and a C-value of 50. Following validation, the *S*. Infantis classifier demonstrated the highest performance (Table 4), achieving 100% sensitivity, 94% specificity, 99% PPV, and 100% NPV. The *S*. Thompson classifier had the second-best performance, with 98% sensitivity, 100% specificity, 100% PPV, and 99% NPV. Finally, the *S*. Montevideo classifier achieved 67% sensitivity, 100% specificity, 100% PPV, and 99% NPV (Table 5). The LDA clustering analysis showed three well-separated groups of spectra: Thompson (red) exhibits more dispersion among the spectra; on the other hand, Infantis (grey) and Montevideo (cyan), both groups of spectra are more concentrated and exhibit well-characterized clusters (Fig. 2).

**TABLE 4:** Confusion matrix for the validation of O:7 classifier^*a*^

		Class predicted				
		Thompson	Infantis	Montevideo	Uncertain	Class recall
**Actual class**	Thompson	103	2	0	0	98% (103/105)
	Infantis	0	691	0	2	99% (691/693)
	Montevideo	0	5	10	0	67% (10/15)
	Class precision	100%	99%	100%		

^*a*^ Accuracy: 99% (804/813).

**TABLE 5:** Values of sensitivity, specificity, PPV, and NPV per serogroup O:7

	Sensitivity	Specificity	PPV	NPV
Thompson	98%	100%	100%	99%
Infantis	100%	94%	99%	100%
Montevideo	67%	100%	100%	99%

### Serogroup O:9 training and validation

**Fig 3:**
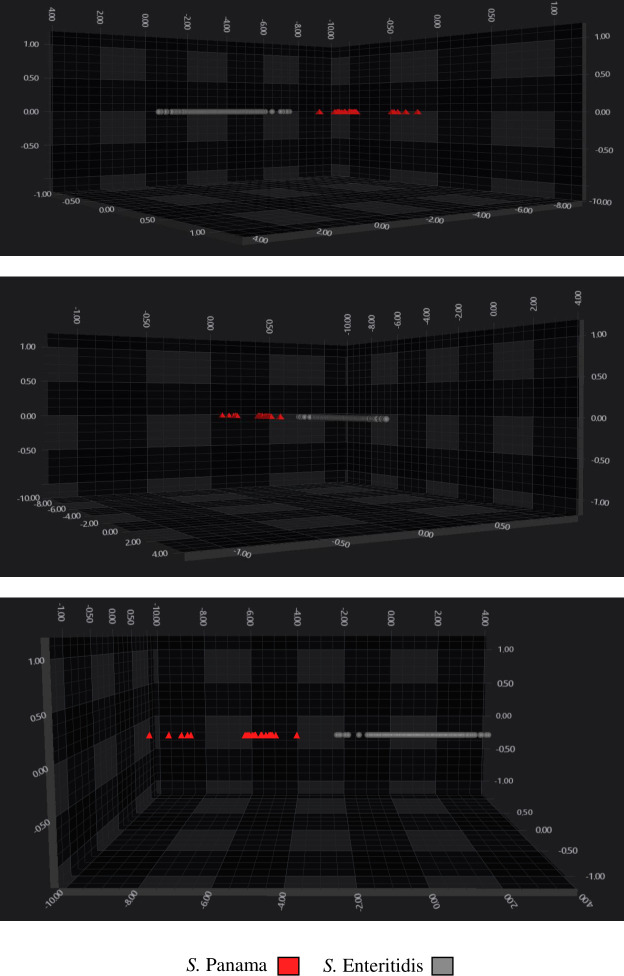
3D scatter plot of the third training performed, showing the distribution of serovars of Salmonella serogroup O:9 species in IR spectral space. A total of 30 PCs were used to create a LDA by serovar. The spectra are colored according to the serovars: red triangles: *S*. Panama; gray circles: *S*. Enteritidis. Every symbol represents a different isolate.

This training utilized 1,393 spectra, employing SVM across a spectral range of 1,300–800 cm^−1^, with parameters set to PC30 and C-value 2. Following validation set analysis, the best-performing classifier was *S*. Enteritidis (Table 6), achieving 100% sensitivity, 80% specificity, 98% PPV, and 100% NPV and *S*. Panama with 80% sensitivity, 100% specificity, 100% PPV, and 98% NPV (Table 7). Analysis of LDA clustering showed a distinct separation of *S*. Enteritidis (grey) and *S*. Panama (red) (Fig. 3).

**TABLE 6:** Confusion matrix for the validation of O:9 classifier^*a*^

		Class predicted			
		Panama	Enteritidis	Uncertain	Class recall
**Actual class**	Panama	20	5	0	80% (20/25)
	Enteritidis	0	289	1	100% (289/290)
	Class precision	100%	98%		

^*a*^ Accuracy: 98% (309/315).

**TABLE 7:** Values of sensitivity, specificity, PPV, and NPV for the validation per serogroup O:9

	Sensitivity	Specificity	PPV	NPV
*S*. Panama	80%	100%	100%	98%
*S*. Enteritidis	100%	80%	98%	100%

## DISCUSSION

*S. enterica* is one of the most significant foodborne pathogens globally, posing substantial threats to both economic stability and public health. Identifying the specific serovar causing a clinical case is critical, as it allows for rapid and effective interventions, thereby mitigating outbreaks and preventing the further spread of infection. In this study, we propose a novel sub-typing method for *Salmonella* based on FT-IR, emphasizing its advantages over traditional or existing approaches. By leveraging discriminatory features identified during training, our classifiers enable the automatic categorization of unidentified samples using a marker model computed from a collection of training spectra. These automated classifiers are designed to seamlessly categorize isolates during spectrum measurement, with a particular focus on *S. enterica* serovars. To achieve our goal of developing a rapid and accurate *Salmonella* typing method, it is important to acknowledge the complexity involved. Differentiation among the approximately 2,600 serovars of *Salmonella* primarily relies on antigenic (phenotypic) distinctions.

As an initial step, the serogroup classifier was validated using the complete set of spectra, demonstrating high performance for each serogroup classifier (Table 1). Based on these results, we can infer that the integrated algorithm exhibits robust discriminatory capacity, effectively clustering spectra according to their serogroups. Similar findings were reported by Campos et al. (31), who evaluated the discriminatory power of FT-IR in distinguishing *Salmonella* serogroups and serovars. By developing algorithms to differentiate various serogroups and serovars within the same serogroup, their study found that the algorithm achieved high performance in discriminating among serogroups O:9, O:7, O:1, O:3, O:19, and O:4. Furthermore, the algorithm demonstrated high typification accuracy in distinguishing between serovars within the same serogroup, indicating its effectiveness in identifying and grouping spectrum features by serovar. It should be noted that different sources of isolates were used (animal, human, and environmental); however, no differences were evident based on the source of isolates. This could be attributed to the fact that most of the serovar analyzed are zoonotic, so it would be expected that there would be no differences between the different sources. Therefore, the possibility of overlap between serovars of the same serogroup could be attributed to structural similarities.

Based on the validation results for the integrated serogroup classifier and building upon previous studies (15, 31, 39), we propose the development of a subtyping algorithm to identify serovars from specific serogroups. This approach leverages the existing serogroup training incorporated into the IR Biotyper software developed by Cordovana et al. (15). This methodology uses a pre-established serogroup classification algorithm as the initial analytical step, followed by a serogroup-specific serovar classification algorithm to enhance typing performance by minimizing variability among trained features.

The best-performing classifiers validations for each serogroup were *S*. Abony (O:4), *S*. Infantis (O:7), and *S*. Enteritidis (O:9). The analysis of the O:4 classifier results reveals three distinct clusters, each representing a serovar: *S*. Abony, *S*. Agona, and *S*. Typhimurium/I,4,[5],12:i:-. Among them, the *S*. Agona cluster is the most clearly definerouped closely together and distinctly separated from the others. In contrast, the *S*. Abony and *S*. Typhimurium/I,4,[5],12:i:- clusters are positioned near each other, obtaining similar results in the training confusion matrix (data not shown). Although this suggests that *S*. Agona has the best-defined cluster, the validation confusion matrix indicates that *S*. Abony had the best overall performance, as it correctly identified all its spectra, despite being closely grouped with *S*. Typhimurium/I,4,[5],12:i:- in the LDA analysis (Fig. 1; Tables 2 and 3). The *S*. Agona classifier, though well-defined in LDA, failed to identify approximately 1% of the total spectra. Meanwhile, the *S*. Typhimurium/I,4,[5],12:i:- classifier had the lowest performance, misclassifying or failing to identify around 8% of its spectra (Fig. 1; Table 2).

The serogroup O:7 classifier validation indicated that the *S*. Thompson and *S*. Infantis classifier was the one that exhibited the best performance, correctly identifying a large number of spectra; this is exhibited in the LDA representation, where the spectra were grouped in a well-identified cluster. Finally, the *S*. Montevideo classifier exhibited the worst performance, misclassifying one-third of the total spectrum, even when the LDA indicated a well-characterized and separated cluster group (Tables 4 and 5; Fig. 2). The small number of *S*. Montevideo isolates and spectra could contribute to the misclassification because the classifier improves predictive performance as the number of training spectra increases isolates.

Analyzing the results obtained for the serogroup O:9 classifier validation, the best performance was obtained by the *S*. Enteritidis classifier, correctly identifying almost 100% of spectra. The performance exhibited by the *S*. Panama classifier is acceptable based on the number of total spectra utilized for the training set, compared with the *S*. Enteritidis classifier (Tables 6 and 7).

As we can evidence in our results, the best performance was not always reached by the classifier that was developed utilizing a higher number of spectra, like some classifiers that exhibited high performance but a lowest number of spectra in the training set; on the other hand, we also evidence that some classifiers, developed with the high number of spectra, also exhibited high performance. This result suggests that the number of required spectra to develop a high-accuracy classifier may differ by each strain type. Additionally, the optimal size for training an SVM model remains uncertain, with no consensus in the existing literature regarding the recommended data size. Recommendations range from hundreds (40) to thousands (28, 29). Based on these suggestions, the number of spectra considered in our study falls within the acceptable range for training and validation data set sizes. Despite this, the investigation could benefit from additional data, as this could further improve the discriminatory power more accurately and robustly by including data processing techniques, such as removal of outlier data and spectra reprocessing (41, 42). We maintain that proper data processing practices, coupled with continuous training and the development of new hierarchical typing algorithms to improve discriminatory power by minimizing the number of trainable features, may have a positive impact on the performance of newly developed classifiers.

### Conclusions

Based on our study, we have learned valuable insights into the application of FT-IR technology for *Salmonella* serotyping. Our findings demonstrate its effectiveness in discriminating between *Salmonella* serogroups O:9, O:7, and O:4, including key serovars such as Typhimurium, Enteritidis, and Infantis. To maximize serovar typing accuracy, we recommend focusing the training set on serovars within the same serogroup and minimizing isolate diversity. In our study, successful serovar identification was achieved, underscoring the importance of adequate training data. We encountered limitations during our research about the number of isolates available by each desired serovar: some of them were represented by under 10 isolates and others above a hundred isolates. Interestingly, the number of isolates by serovar is not directly related to the accuracy of the developed classifier.

Accurate identification of *Salmonella* serovars has far-reaching implications across multiple sectors, including clinical diagnostics, epidemiological surveillance, and various branches of the food industry. By improving the speed and accuracy of serovar identification, advanced technologies like the IR Biotyper provide solutions to long-standing challenges in public health and industrial settings.

To maximize the impact of these technologies, we recommend ongoing validation, training data set updates, and adaptation to local and emerging *Salmonella* serovars. By doing so, advanced serotyping methods can remain reliable, scalable, and effective in addressing public health challenges, supporting surveillance efforts and improving food safety practices across industries.

These applications underscore the versatility and potential of advanced serotyping technologies to bridge the gap between scientific innovation and practical solutions in diagnostics, surveillance, and industry.

## SUPPLEMENTAL MATERIAL

Table S1 spectrum.00159-25-s0001.xlsx Salmonella O:4, O:7, and O:9 isolates by different sources with serovar and serogroup classification.

ASM does not own the copyrights to Supplemental Material that may be linked to, or accessed through, an article. The authors have granted ASM a non-exclusive, world-wide license to publish the Supplemental Material files. Please contact the corresponding author directly for reuse.
